# Overlap Syndrome of Ankylosing Spondylitis and Mixed Connective Tissue Disease in a Male Patient

**DOI:** 10.7759/cureus.105304

**Published:** 2026-03-16

**Authors:** Sofia Kada, Saadia Ait Malek, Erraoui Mariam, Imad Ghozlani

**Affiliations:** 1 Rheumatology, Mohammed VI University Hospital, Agadir, MAR; 2 Cartilage and Bone (CARBONE) Research Team, Research Laboratory of Innovation in Health Sciences (LARISS), Faculty of Medicine and Pharmacy of Agadir, Ibn Zohr University, Agadir, MAR; 3 Rheumatology, Oued Eddahab Military Hospital, Agadir, MAR

**Keywords:** ankylosing spondylitis, mixed connective tissue disease, overlap syndrome, rare, u1rnp

## Abstract

Spondyloarthritis (SPA) is a chronic systemic inflammatory disease predominantly affecting young men, with axial and peripheral involvement and extra-articular manifestations, including uveitis, inflammatory bowel disease, and psoriasis. Mixed connective tissue disease (MCTD) is a rare, heterogeneous autoimmune disorder primarily affecting women of reproductive age. It is characterized by circulating autoantibodies leading to immune-mediated inflammation and progressive tissue damage. The coexistence of these two conditions is rarely reported in the literature.

The present study reports the case of a 25-year-old male patient who had ankylosing spondylitis (AS) for four years and had been receiving diclofenac for six months, followed by ketoprofen at a dose of 150 mg/day and sulfasalazine at 2 g daily. The patient presented with severe pain and high disease activity after three years of remission under treatment. Immunological testing revealed positivity for anti-U1 RNP antibodies (11 UA). Based on these findings, a diagnosis of MCTD was established. The patient received high-dose intravenous corticosteroid therapy, with a significant clinical and biological response.

## Introduction

Spondyloarthritis (SPA) is a chronic inflammatory disease that mainly affects young men, causing back pain, arthritis, and sometimes extra-articular features such as uveitis, sacroiliitis, colitis, or psoriasis. HLA-B27 is the most common genetic association. Mixed connective tissue disease (MCTD) is a rare overlap syndrome combining features of lupus, systemic sclerosis, and polymyositis, with anti-U1 RNP antibodies driving immune-mediated tissue damage. The coexistence of SPA and MCTD is unusual [1]. We report the case of a 25-year-old man with a four-year history of ankylosing spondylitis (AS) who was diagnosed with MCTD.

## Case presentation

**Figure 1:**
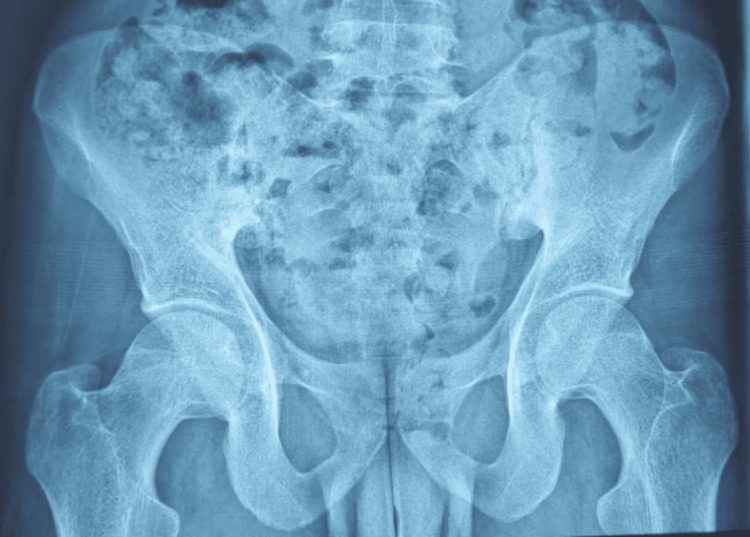
Pelvic radiography revealing bilateral stage III sacroiliitis: blurred sacroiliac joint margins, sclerosis of both iliac sides, and mild irregularity of the ischial margins

Our patient is a 25-year-old man with no significant past medical history, except for chronic smoking. Symptoms began in 2021 with inflammatory back pain, predominantly at the thoracolumbar junction, associated with alternating inflammatory buttock pain. The disease course was marked by the subsequent onset of inflammatory arthralgia involving large and medium-sized joints, accompanied by joint swelling. Pelvic radiography revealed bilateral stage III sacroiliitis (Figure 1).

**Figure 2:**
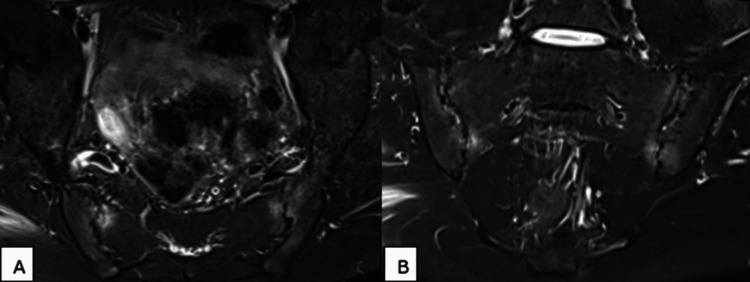
(A) Axial and (B) coronal MRI in STIR sequence of the sacroiliac joints revealing subchondral bone marrow edema with associated erosions

MRI of the sacroiliac joints showed extensive subchondral bone marrow edema on both the iliac and sacral sides, appearing as hyperintensity on short tau inversion recovery (STIR) sequences, with associated erosions predominantly on the iliac aspect (Figure 2).

The patient was positive for HLA-B27. Based on these findings, a diagnosis of AS was established. Initial treatment consisted of diclofenac 150 mg/day for six months, followed by ketoprofen 150 mg/day until admission. The patient did not report any extra-articular manifestations, including psoriasiform skin lesions, gastrointestinal symptoms, or sicca features.

At admission, pain intensity was rated at 90 mm on the visual analogue scale (VAS) and fatigue at 80 mm. Physical examination revealed a painful cervical spine with restricted lateral flexion. Lumbar examination showed loss of lordosis and limited mobility, with a modified Schöber index of 13 cm. Sacroiliac joint examination demonstrated pain on pressure, with all provocative maneuvers positive.

Laboratory testing revealed a marked inflammatory syndrome (erythrocyte sedimentation rate (ESR), 45 mm/h; C-reactive protein (CRP), 119.81 mg/L; Table 1) and high disease activity, with an Ankylosing Spondylitis Disease Activity Score (ASDAS-CRP) of 4.36 and a Bath Ankylosing Spondylitis Disease Activity Index (BASDAI) of 7.1, consistent with highly active SPA. The ASDAS-CRP score ranges from 0.6 to infinity, with higher values indicating greater disease activity, and the BASDAI score ranges from 0 to 10, with higher scores reflecting increased disease activity [2,3].

The patient also presented with hand edema, Raynaud's phenomenon associated with microvascular abnormalities on capillaroscopy, and diffuse myalgia with elevated creatine phosphokinase (CPK) levels. This prompted us to request an ENA panel, which revealed positive anti-U1-RNP antibodies. Anti-DNA antibodies were negative, and no complement consumption was observed (Table 1).

**Table 1:** Laboratory test findings, with reference ranges ESR: erythrocyte sedimentation rate, CRP: C-reactive protein, CPK: creatine phosphokinase, A-U1 RNP: antibodies to U1 ribonucleoprotein particle, C3: complement component 3, C4: complement component 4, CH50: total complement hemolytic activity, anti-DNA: anti-double-stranded DNA antibodies.

Laboratory tests	Results	Reference ranges
ESR	45 mm/1st hour	<20 mm/1st hour
CRP	119.81 mg/L	<6 mg/L
CPK	350 UI/L	40-200 U/L
A-U1 RNP	11 UA	<5 UA
C3	1.5 g/L	0.9-1.8 g/L
C4	2 g/L	0.10-0.40 g/L
CH50	92 U/mL	50-150 U/mL
Anti-DNA	12 UI/mL	<30 UI/mL

The patient was treated with intravenous corticosteroid pulses, resulting in marked clinical and biological improvement, and was considered a candidate for anti-TNF therapy.

## Discussion

SPA and MCTD are considered clinically distinct conditions. However, some case reports have suggested a possible association between the two, either due to immune system dysregulation or as a result of treatments such as sulfasalazine and biologic agents like TNF inhibitors. Sulfasalazine has been associated with the induction of antinuclear antibodies (ANA) and the development of drug-induced systemic lupus erythematosus (SLE) [1].

Previous reports have described the coexistence of SPA and MCTD, particularly with Sjögren’s syndrome [4]. In this context, our case illustrates an uncommon association between AS and MCTD, highlighting the potential for overlap between these conditions. The first reported case was described in 1998 by Galluzzo et al. [5], and several additional cases were subsequently reported by other authors, notably Dharmapalaiah et al. [6].

Our patient did not exhibit the classic manifestations described by Tanaka et al. [7], such as hand edema, myositis, or mechanic’s hands. However, he presented with synovitis, fever, lymphadenopathy, and a positive ENA panel revealing anti-U1 RNP antibodies, supporting the diagnosis of MCTD.

Treatment strategies for MCTD are not widely standardized. In the study by Hajas et al., 78% of patients received high-dose steroids, around 75% were treated with methotrexate or cyclophosphamide, and 15% received anti-TNF therapy [8].

Similarly, Cappelli et al. reported that over half of the patients were on immunosuppressants, more than 80% required glucocorticoids, and nearly half were treated with antimalarial medications [9].

Our patient received intravenous corticosteroid pulses, which led to significant clinical and laboratory improvement, and he was considered a candidate for biologic therapy, particularly anti-TNF agents.

## Conclusions

In summary, although SPA and MCTD are usually considered separate conditions, their coexistence has been documented, though it remains rare. These overlapping cases highlight how autoimmune diseases can sometimes blur the boundaries between established categories. Patients may present with unusual or atypical symptoms, so careful clinical assessment and targeted immunological testing are essential. Treatment should be individualized, often involving immunosuppressive medications and corticosteroids to control inflammation, depending on the organs involved. Recognizing this association can help clinicians provide timely and effective care for patients with complex rheumatologic presentations.
